# Human papillomavirus (HPV) vaccination of girls in Germany. Results of the cross-sectional KiGGS Wave 2 study and trends

**DOI:** 10.17886/RKI-GBE-2018-102.2

**Published:** 2018-12-12

**Authors:** Christina Poethko-Müller, Nina Buttmann-Schweiger, Anja Takla

**Affiliations:** 1 Robert Koch Institute, Berlin, Department of Epidemiology and Health Monitoring; 2 Robert Koch Institute, Berlin, Department of Infectious Disease Epidemiology

**Keywords:** HUMAN PAPILLOMAVIRUS, HPV, VACCINATION COVERAGE, TREND, HEALTH MONITORING, KIGGS

## Abstract

Since 2007, the Standing Committee on Vaccination (STIKO) has recommended that all girls receive vaccinations against the human papillomavirus (HPV) in order to reduce the disease burden of cervical cancer. Persistent infections with high-risk HPV subtypes increase a woman’s risk of developing cancer. In the second wave of the German Health Interview and Examination Survey for Children and Adolescents (KiGGS Wave 2, 2014-2017), 42% of 2,958 11- to 17-year-old girls reported that they had received at least one HPV vaccination, and 31.4% reported a full HPV vaccination. 45.3% of 14- to 17-year-old girls reported a complete series of HPV vaccinations. Compared to the figures reported in KiGGS Wave 1 five years ago, HPV vaccination coverage has therefore remained stable. A vaccination coverage below 50% in girls is too low to exploit the potential of HPV vaccination to reduce cervical cancer rates in Germany.

## Introduction

The human papillomavirus (HPV) is sexually transmitted. Without vaccination, over two thirds of all women will contract HPV during the course of their lives [1]. HPV is subdivided into high and low risk types. While high risk types can lead to cancer (carcinoma), low risk types are known to cause genital warts. Types 16 and 18 are the most important high risk types that play a critical role in the development of cervical carcinoma and cause around 70% of all cancers of the cervix uteri globally.

Factors such as a high virus burden and the carcinogenicity of a specific virus type further increase the risk of cancer. Smoking or an HIV infection increase the risk of cancer as well. In most cases, however, an HPV infection is cleared without further consequences. The infection persists only in about one in ten women for one to two years and increases the risk of developing cancer [2]. In 2014, around 4,540 women were diagnosed with cervical cancer in Germany and around 1,540 women died of the disease [3]. Over 50,000 women annually are estimated to undergo cervical surgery (conisation) to treat HPV-related precancerous stages [4].

Since 2007, the Standing Committee on Vaccination (STIKO) has recommended HPV vaccination for all girls as a means of reducing the burden of cervical cancer. Until 2014, a series of three vaccinations was generally recommended for 12- to 17-year-old girls. In August 2014, the STIKO lowered the recommended age for vaccination of girls to 9- to 14-years and advised two instead of three vaccinations for this age group for full immunisation [5]. Catch-up vaccinations are recommended up to the age of 17 years [6].

**KiGGS Wave 2**

Second follow-up to the German Health Interview and Examination Survey for Children and Adolescents

**Data owner:** Robert Koch Institute

**Aim:** Providing reliable information on health status, health-related behaviour, living conditions, protective and risk factors, and health care among children, adolescents and young adults living in Germany, with the possibility of trend and longitudinal analyses

**Study design**: Combined cross-sectional and cohort study

**Cross-sectional study in KiGGS Wave 2**

**Age range:** 0-17 years

**Population:** Children and adolescents with permanent residence in Germany

**Sampling:** Samples from official residency registries - randomly selected children and adolescents from the 167 cities and municipalities covered by the KiGGS baseline study

**Sample size:** 15,023 participants

**KiGGS cohort study in KiGGS Wave 2**

**Age range:** 10-31 years

**Sampling:** Re-invitation of everyone who took part in the KiGGS baseline study and who was willing to participate in a follow-up

**Sample size:** 10,853 participants

**KiGGS survey waves**

► KiGGS baseline study (2003-2006), examination and interview survey
► KiGGS Wave 1 (2009-2012), interview survey
► KiGGS Wave 2 (2014-2017), examination and interview survey

More information is available at www.kiggs-studie.de/english

The second wave of the German Health Interview and Examination Survey for Children and Adolescents (KiGGS Wave 2, 2014-2017) now provides data on HPV vaccination coverage based on a population-based representative sample. These data complement the calculation of vaccination coverage conducted by the Robert Koch Institute (RKI) in cooperation with the Associations of Statutory Health Insurance Physicians (ASHIPs) which is based on the analysis of health insurance claims data (ASHIP vaccination monitoring project) and permit the inclusion of privately insured girls as well as an analysis of further indicators for differences in vaccination uptake. A comparison with data collected five years ago in KiGGS Wave 1 allows for a trend analysis in HPV vaccination coverage and their determinants.

## Indicator

KiGGS is part of the health monitoring system at the RKI and includes repeated cross-sectional surveys of children and adolescents aged 0 to 17 years (KiGGS cross-sectional study) that are representative for Germany. The KiGGS baseline study (2003-2006) was conducted as an examination and interview survey, the first follow-up study (KiGGS Wave 1, 2009-2012) as a telephone-based interview survey and KiGGS Wave 2 (2014-2017) as an examination and interview survey. A detailed description of the methodology used in KiGGS Wave 2 can be found in New data for action. Data collection for KiGGS Wave 2 has been completed in issue S3/2017 as well as in KiGGS Wave 2 cross-sectional study – participant acquisition, response rates and representativeness in issue 1/2018 of the Journal of Health Monitoring [7, 8].

HPV vaccination coverage was surveyed in KiGGS Wave 2 through the answers of 11- to 17-year-old girls to a written questionnaire. It contained the question: ‘Have you been vaccinated against human papillomavirus (HPV) to protect yourself from cervical cancer?’ Respondents answering ‘yes’ were asked how many vaccinations they had received (‘How many HPV vaccinations have you received so far?’) and how old they had been when they received their initial vaccination (‘At what age were you first vaccinated against HPV?’).

The analyses are based on data from 2,958 girls aged 11 to 17 with valid information on HPV vaccination. The proportion of missing values on HPV vaccination in the group of 11- to 12-year-old girls is high (20.3%) compared with the group of 13- to 17-year-old girls (10.6%). The analyses on the completed series of HPV vaccinations is based on the data collected from 2,916 girls (98.6%) because 42 girls did not provide answers to the respective subquestions. Results are presented as prevalences (frequencies) for at least one vaccination and for the completed vaccination series according to current STIKO recommendations. A completed series of vaccinations is defined as two vaccinations if started until the age of 14 years or three vaccinations if started at 15 years or older. The analysis did not consider whether there was a minimum interval between vaccinations of six months between the two vaccinations (or five months, according to current recommendations). The tables show prevalences stratified according to age, residence in East or West German federal states and socio economic status (SES) [9]. To analyse trends, the information on HPV vaccinations that respondents self-reported in KiGGS Wave 1 were recalculated following the current STIKO recommendations to provide an identical indicator, with the exception of the different surveying method (telephone interview vs. written questionnaire). Due to the high number of non-respondents among girls less than 14 years of age, trends were calculated limited to 14- to 17-year-old girls, in line with the approach chosen to assess KiGGS Wave 1 results [10].

The calculations were carried out using a weighting factor that corrects deviations within the sample from the population structure with regard to regional structure (rural/urban area), age (in years), gender, federal state (as of 31 December 2015), German citizenship (as of 31 December 2014) and the parents’ level of education (Microcensus 2013 [11]). P-values to demonstrate temporal trends relative to KiGGS Wave 1 were based on age-standardised prevalences (as of 31 December 2015). This article considers prevalences with 95% confidence intervals (95% CI). Prevalences are estimates and their precision can be assessed through the use of confidence intervals - wide confidence intervals indicating a greater statistical uncertainty in the results. A statistically significant difference between groups is assumed if the corresponding p-value is less than 0.05 under consideration of weighting and survey design.

## Results and discussion

**Figure 1:**
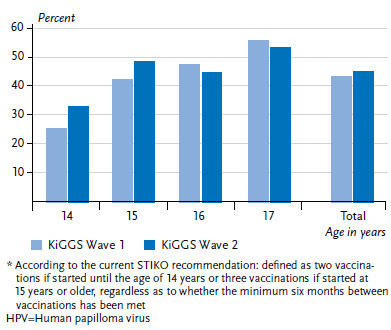
Prevalence of self-reported complete HPV vaccination series* for 14- to 17-year old girls according to age in KiGGS Wave 1 (n=1,326) and KiGGS Wave 2 (n=1,668) Source: KiGGS Wave 1 (2009-2012), KiGGS Wave 2 (2014-2017)

In KiGGS Wave 2, 42.0% of 11- to 17-year-old girls reported having received at least one HPV vaccination. The proportion of girls who have received at least one HPV vaccination increases with age. Whereas in the group of 11- to 13-year-old girls, 20.6% had received at least one vaccination, this proportion rises to 55.5% among 14- to 17-year-olds. Less than one third of 11- to 17-year-old girls has received the complete vaccination series. The proportion of girls with complete HPV vaccination also increases with age. Only 9.3% of 11- to 13-year-old girls, but 45.3% of 14- to 17-year-old girls have received a complete HPV vaccination series (Table 1). Figure 1 contains a stratification by single year of age. There are significant differences between girls who live in East and West German federal states. Whereas the vaccination coverage of 11- to 17-year-old girls in the Eastern German federal states is 43.6%, a considerably lower proportion of girls in Western Germany has received the complete HPV vaccination series (29.5%) (Table 1). Socio economic status did not influence HPV coverage.

Compared to the girls interviewed by telephone five years ago for KiGGS Wave 1, there are no apparent differences in vaccination coverage for 14- to 17-year-old girls. The proportion of fully vaccinated adolescent girls was 43.7% in KiGGS Wave 1 and therefore only slightly lower than the currently surveyed prevalences (45.3%) (Figure 1). The slight rise in the proportion of fully vaccinated younger girls might already indicate a tendency towards earlier vaccination, which would be in line with the STIKO recommendation of August 2014 to lower the age of vaccination to 9- to 14-year-old girls [5]. Whereas in KiGGS Wave 1, the median age at initial vaccination for 12- to 17-year-old girls was 14 years and 2 months, the median age of the 12- to 17-year old girls surveyed in KiGGS Wave 2 for their first HPV vaccination was 13 years and 9 months and therefore 5 months earlier than in KiGGS Wave 1 (data not shown).

When comparing the HPV vaccination coverage surveyed in KiGGS Wave 2 between 2014 and 2017 with the vaccination coverage calculated for 15-year-old girls in 2015 based on the ASHIP vaccination monitoring project [12], a higher proportion of girls in KiGGS Wave 2 (48.7% of 15-year-old girls) have received a complete series of HPV vaccination than seen in the ASHIP vaccination monitoring project data among girls of that age (31.3 %). The relative difference between East and West Germany is similar in both surveys. Both studies apply current criteria for a complete series of HPV vaccination: two vaccinations are considered sufficient if the first dose is administered before the age of 14 years. However, only ASHIP data takes into account an important additional condition for the two-dose schedule: a minimum of six months between the first and second vaccination dose is required. Consideration of this requirement might have contributed to the lower vaccination coverage reported by the ASHIP vaccination monitoring project. Further possible explanations could be recall bias in self-reporting on vaccinations as well as providing socially desired answers. Moreover, it can be assumed that participants in health studies are more aware of their health and that their vaccination coverage might differ from the general population (selection bias). This is undermined by the prevalence of participation in adolescent routine health screening examinations (J1 examination), indicating a higher level of utilization of health services by girls participating in KiGGS. For 15-year-old girls who participated in KiGGS Wave 1 (2009 – 2012), the rate was significantly higher than the prevalences calculated based on ASHIP data [12] for 15-year-old girls (60.1% vs 50.1%). It must therefore be assumed that the HPV vaccination coverage surveyed in KiGGS Wave 2 overestimates the actual vaccination coverage in German girls.

In the years following the HPV vaccination recommendation for girls, use and risk profile of HPV vaccination has been discussed widely and critically in the general public [13], leading to a hesitant or negative attitude towards HPV vaccination in parts of the population. KiGGS Wave 1 results still reflected those attitudes by showing clear differences in HPV vaccination coverage according to socioeconomic status [10]. KiGGS Wave 2 no longer shows an association between HPV vaccination coverage and socioeconomic status. Moreover, in a current survey of the German Federal Centre for Health Education (BZgA), nearly 80% of 16- to 20-year-old women considered HPV vaccination as ‘(particularly) important’ [14]. At the same time, in a Facebook-based survey among young women on HPV vaccination, safety concerns were the most common reason given for not receiving the vaccination [15]. Besides lack of information on the safety profile of the vaccination, another reason for the relatively low vaccination coverage in the age group of 9- to 17-year-old girls could be structural issues such as the significantly lower frequency of physician consultations compared to the groups of infants and toddlers. A good opportunity for counselling on HPV vaccination for this age group is the J1 early detection examination for 12-to 14-year-old adolescents. However, in spite of great regional differences, only 50% of adolescents in Germany participate [16]. An analysis of ASHIP data showed that 12-year-old girls that participated in the J1 examination were seven times more likely to have received at least one HPV vaccination [12]. This positive association was also clearly evident in KiGGS Wave 1 data for the age group of 14- to 17-year-old girls [10].

HPV vaccination coverage surveyed in KiGGS indicates that only about half of all girls or their parents/legal guardians, respectively, decide for a vaccination to protect them against HPV infections. The preventive potential of HPV vaccination against cervical cancer is so far not being fully exploited. In addition to the direct, individual protection, with a sufficiently high vaccination coverage of around 80% protection through herd immunity can be observed [17], also allowing for protection of unimmunized women in the community. In the summer of 2018, STIKO extended the HPV vaccination recommendation also to 9- to 14-year-old boys [18]. The additional vaccination of boys will most likely lead to an increase of the positive vaccination effects, as more young people will be protected directly and indirectly through herd immunity against HPV infections and HPV-associated tumors.

## Key statements

- 45.3% of 14- to 17-year-old girls have received a complete series of HPV vaccinations.
- Vaccination coverage of girls in East German federal states is significantly higher than in the West German federal states.
- 12- to 17-year-old girls received their first HPV vaccination on average at the age of 13 years and nine months which is five months earlier than in KiGGS Wave 1 five years ago.

## Figures and Tables

**Table 1:** Prevalences of at least one single HPV vaccination dose (n=2,958 girls) and self-reported complete HPV vaccination series* (n=2,916 girls) according to age, socioeconomic status and place of residence Source: KiGGS Wave 2 (2014-2017)

At least on HPV vaccination dose			Complete HPV vaccination series*		
%		(95 % CI)	%		(95% CI)
**Girls (total)**	42.0	(39.1-45.1)	**Girls (total)**	31.4	(28.9-33.9)
**Age group**			**Age group**		
11-13 Years	20.6	(17.7-23.8)	11-13 Years	9.3	(7.5-11.5)
14-17 Years	55.5	(51.7-59.4)	14-17 Years	45.3	(41.8-48.9)
**Socioeconomic status**			**Socioeconomic status**		
Low	42.0	(35.5-48.8)	Low	30.1	(24.5-36.4)
Medium	42.6	(39.0-46.2)	Medium	32.2	(29.3-35.2)
High	41.0	(36.8-45.4)	High	31.6	(27.9-35.5)
**Place of residence**			**Place of residence**		
East German federal states	55.6	(50.7-60.4)	East German federal states	43.6	(39.1-48.2)
West German federal states	40.0	(36.7-43.3)	West German federal states	29.5	(26.9-32.2)

* According to the current STIKO recommendation: defined as two vaccinations if started until the age of 14 years or three vaccinations if started at 15 years or older, regardless as to whether the minimum six months between vaccinations has been met

HPV=Human papilloma virus
