# Corrigendum: Human papillomavirus (HPV) vaccination of girls in Germany. Results of the cross-sectional KiGGS Wave 2 study and trends

**DOI:** 10.25646/6804

**Published:** 2019-01-31

**Authors:** Christina Poethko-Müller, Nina Buttmann-Schweiger, Anja Takla

Poethko-Müller C, Buttmann-Schweiger N, Takla A (2018)

Journal of Health Monitoring 3(4): 79-86.

DOI 10.17886/RKI-GBE-2018-102

An earlier version of this Fact sheet gave the following incorrect figures on page 81: ‘Whereas the vaccination coverage of 11- to 17-year-old girls in the Eastern German federal states is 55.6%, a considerably lower proportion of girls in Western Germany has received the complete HPV vaccination series (40.0%) (Table 1).’

These figures incorrectly reported the coverage of ‘at least one single HPV vaccination dose’.

The correct sentence reads: ‘Whereas the vaccination coverage of 11- to 17-year-old girls in the Eastern German federal states is 43.6%, a considerably lower proportion of girls in Western Germany has received the complete HPV vaccination series (29.5%) (Table 1).’

The corrected version of the article is available at DOI 10.17886/RKI-GBE-2018-102.2

